# Protective effects of the *Terminalia bellirica* tannin-induced Nrf2/HO-1 signaling pathway in rats with high-altitude pulmonary hypertension

**DOI:** 10.1186/s12906-023-03981-2

**Published:** 2023-05-06

**Authors:** Salamaiti Aimaier, Yang Tao, Fang Lei, Zhang Yupeng, Shi Wenhui, Ainiwaer Aikemu, Dilinuer Maimaitiyiming

**Affiliations:** 1 Heart Center, The First Affiliated Hospital of Xinjiang Medical University, Urumqi, 830054 China; 2 College of pharmacy, Xinjiang Medical University, Urumqi, 830011 China; 3 Central Laboratory, Xinjiang Medical University, Urumqi, 830011 China; 4 Key Laboratory of Special Environmental Medicine of Xinjiang, General Hospital of Xinjiang Military Region of PLA, Urumqi, 830000 China

**Keywords:** *Terminalia bellirica* (Gaertn.) Roxb, High-altitude pulmonary hypertension, Tannins, Nrf2, HO-1

## Abstract

**Background:**

Oxidative stress and endothelial cell dysfunction induced by high-altitude hypoxia have important roles in the pathological process of high-altitude pulmonary hypertension (HAPH). Tannins present in *Terminalia bellirica* (Gaertn.) Roxb. (TTR) have pharmacological activities that produce oxidation resistance and exert anti-inflammatory effects. Whether TTR exerts a protective effect on HAPH remains unknown.

**Methods:**

A rat model of HAPH was established. The mean pulmonary arterial pressure (mPAP) of the animals was measured, the serum levels of SOD, MDA, and GSH-Px were measured using ELISA, and the expression of Bax, Bcl-2, Nrf2, and HO-1 proteins in the lung tissue of each group of rats was measured using Western blotting. Pathological changes in the lung tissue were also observed. A model of damage to H_2_O_2_-induced pulmonary artery endothelial cells (PAECs) was generated, and cell proliferation was measured using CCK-8 assays. Flow cytometry was used to measure ROS levels in PAECs. Western blotting was used to detect the expression of Bax, Bcl-2, Nrf2, and HO-1 proteins in PAECs.

**Results:**

The hemodynamic and pathologic findings showed that the mPAP of HAPH rats increased markedly, and the vascular wall thickness increased (*P* < 0.05). TTR reduced mPAP, alleviated or slowed pulmonary arterial remodeling, increased GSH-Px and SOD activity, lowered the level of MDA (*P* < 0.05), and downregulated the expression of Bax in the lung tissues of HAPH rats, while the expression of Bcl-2, Nrf2, and HO-1 was upregulated (*P* < 0.05). The results of the cell experiments showed that TTR inhibited H_2_O_2_-induced PAEC apoptosis and ROS production (*P* < 0.05), downregulated the expression of Bax in PAECs, and upregulated the expression of Bcl-2, Nrf2, and HO-1 (*P* < 0.05).

**Conclusion:**

The results suggest that TTR reduces pulmonary arterial pressure, decreases oxidative stress during HAPH, and exerts protective effects in rats with HAPH and that its mechanism of action is related to regulation of the Nrf2/HO-1 signaling pathway.

**Supplementary Information:**

The online version contains supplementary material available at 10.1186/s12906-023-03981-2.

## Introduction

Mean pulmonary arterial pressure (mPAP) refers to arterial pressure in the resting state. HAPH refers to a mPAP of ≥ 25 mmHg, increased by high-altitude hypoxia at elevations over 2500 m above sea level; mPAP values in this range fall within the scope of type III pulmonary hypertension [1, 2]. Long-term elevation of PAP can cause high-altitude pulmonary edema and heart disease and may even lead to death as the disease progresses.

The pathogenesis of HAPH has not been fully elucidated. Oxidative stress and endothelial cell dysfunction, as well as proliferation of pulmonary artery smooth muscle cells (PASMCs), are the main pathological changes that promote the progression of HAPH [3]. When experiencing high-altitude hypoxia, the body responds to oxidative stress; the concentrations of markers of oxidative damage, such as methionine sulfoxide, protein carboxyl groups, advanced oxidation protein products, and malondialdehyde [4, 5], increases, and the concentration of the endogenous antioxidants superoxide dismutase (SOD) and glutathione decreases. Oxidative stress is one of the main factors that produce endothelial injury and vascular wall remodeling [6, 7]. The oxidative stress response may damage blood vessels, undermine endovascular homeostasis, increase cellular Ca^2+^ concentrations, and result in the degradation of proteins, lipids, and DNA [8, 9]. During hypoxia, superoxide anions formed in the body alleviate NO-mediated vasodilation by producing peroxynitrites [10, 11]. Thus, treatment of antioxidant stress has potential for the treatment of HAPH. Nuclear factor E2-related factor 2 (Nrf2) plays a crucial role in oxidation resistance. Active Nrf2 upregulates downstream targets, enhances resistance to oxidation, alleviates endothelial cell disorders, and contributes to vascular remodeling. The Nrf2/antioxidant response element (ARE) signaling pathway has been shown to regulate the downstream target heme oxygenase 1 (HO-1) and thereby to induce antioxidant stress and protect cells [12]. Previous studies have shown that the expression of HO-1 in the lung tissues of individuals with severe HAPH is clearly downregulated compared with its expression in individuals without HAPH, and upregulation of HO-1 has been shown to inhibit the progression of pulmonary hypertension [13]. Therefore, the Nrf2/HO-1 signaling pathway is of great significance and is becoming a promising target in the treatment of HAPH.

A significant proportion of people in developed countries use Complementary and alternative medicines(CAM) medical services, as well as in developing countries.There is evidence that more and more people with chronic diseases are using CAM to manage their chronic diseases. [14–16].Chinese medicinal herbs have been widely studied because they are an abundant resource with a long history of use. *Terminalia bellirica* (Gaertn.) Roxb. (TTR) is used in Tibetan medicine [17]; its dried ripe fruit is rich in tannins, including ellagic acid, tannic acid, and corilagin, and it has pharmacological activities such as anti-inflammatory effects and oxidative stress resistance [18–20]. San-guo Tang, a Tibetan medicine consisting of TTR, *Terminalia chebula* Retz., and *Phyllanthus emblica* Linn., was shown to have definite therapeutic effects in individuals with high-altitude diseases [21]. Previous studies have shown that TTR extract exerts antioxidant activity by breaking free radical chain reactions, scavenging free radical initiators, reducing ROS concentrations and chelating transition metals that help catalyze free radical production in vivo and ex vivo [22–26]. However, whether TTR improves HAPH by regulating the Nrf2/HO-1 signaling axis needs to be further studied.

In the present study, a rat model of HAPH and a cell model of H_2_O_2_-induced pulmonary artery endothelial cell (PAEC) injury were established and used to explore the protective effects of TTR against HAPH and its antioxidant effects via the Nrf2/HO-1 signaling pathway.

## Materials and methods

### Target animals and cells

Sixty male specific-pathogen-free Sprague‒Dawley (SD) rats (mean body weight 200 ± 30 g) were provided by the Experimental Animal Center of Xinjiang Medical University (Experimental Animal Production Permit No.: SCXK(Xin)2018-0003. The PAEC rat strain was donated by the Collaborative Innovation Center Laboratory of Xinjiang Medical University.

### Reagents

TTR extract (purity > 50%) was prepared by our laboratory. Sildenafil was purchased from Pfizer Pharmaceuticals Limited. SOD, glutathione peroxidase (GSH-Px), and malondialdehyde (MDA) detection kits were obtained from Nanjing Jiancheng Bioengineering Institute. Enhanced RIPA lysate, broad-spectrum phosphatase inhibitor, and broad-spectrum protease inhibitor were obtained from Boster Bio Co., Ltd. BCA protein quantitation kits were purchased from Mei5 Biotech Co., Ltd. The following antibodies were used in the experiments: anti-Nrf2 (Wanleibio), anti-HO-1, anti-Bax, and anti-Bcl-2 (Abcam, USA), anti-β-actin (Beijing Affinity Biosciences), and HRP-conjugated AffiniPure goat anti-rabbit IgG (H + L) (Proteintech, Cat No. SA00001-2). Fetal calf serum was obtained from Gibco, USA. Primary endothelial cell basal culture medium, cell growth factor, and penicillin‒streptomycin double antibiotic were obtained from iCell Bioscience Inc. Pancreatin (0.25%) was purchased from HyClone, USA. Dimethyl sulfoxide was purchased from BIOFROXX, USA. Cell proliferation and toxicity detection kits were obtained from Bioss Biotech; apoptosis detection kits were obtained from USA BD, and reactive oxygen species (ROS) detection kits were obtained from Beijing Solarbio.

### Instruments

The following equipment was used in this study (manufacturers are listed in parentheses): ELISA reader (Model No. 352, Labsystems Multiskan MS, Finland); microplate washer (Model No. AC8, Thermo Lab-systems, Finland); water-jacketed thermostatic incubator (Model No. GNP-9080, Shanghai Xiangfan Instruments Co., Ltd., China); small rodent ventilator (Model No. ks606731, Beijing Kesijia Technology Co., Ltd., China); electrophysiology recording system (Model BL-420 S, Chengdu Techman Electronics Co., Ltd., China); small vertical electrophoresis transfer system (Mini-PROTEAN Tetra Cell, Bio-Rad, USA); chemiluminescence gel imager (FluorChem E, Protein Simple, USA); Northwest Special Artificial Environment Laboratory (Model DY-2, Guizhou Fenglei Aviation Ordnance Co., Ltd., China); low-temperature high-speed centrifuge (Allegra-64R, Beckman Coulter, USA); MLS-3020U autoclave (Model SX-500, Sanyo, Japan); inverted fluorescence microscope (Model No. DMi8 automated, Leica, Germany); full-wavelength microplate reader (Thermo Multiskan GO, Thermo, USA); and flow cytometer (FACSCalibur, BD, USA).

### Preparation of TTR

To two kilograms of *Terminalia bellirica* (Gaertn.) Roxb., a 20-fold excess of 70% ethanol was added; the mixture was refluxed and extracted twice, each time for 2 h. The extracts were combined, concentrated under reduced pressure, and set aside. The residual material was extracted twice with 20 volumes of water for 2 h each time. The concentrated ethanol extract was combined with the water extraction solution, and the mixture was concentrated under reduced pressure and used as a resin purification sample solution for backup.

The concentration of the sample solution was 71.2 mg/mL. The sample was passed over HPD600 resin (the volume of the sample was 3 bed volumes (BV)) at a flow rate of 1 BV·h^− 1^. The bound material was eluted with 3 BV of water; the resin was then eluted with 5 BV of 50% ethanol at a flow rate of 3 BV·h^− 1^. The eluents were combined, concentrated under reduced pressure, and freeze-dried to obtain TTR. The phosphomolybdate tungstic acid casein method was used to determine the total tannin content of the material.

### HAPH modeling and intervention

We use methods that have been described in the literature to establish a HAPH rat model in a simulated plateau environment [4].

Fifty HAPH rats were randomly divided into five groups: a model group (administered normal saline, 1 mL/100 g/day), a sildenafil group (administered sildenafil tablets, 30 mg/kg/day), and TTR-H, TTR-M, and TTR-L groups (the animals in these groups were given TTR at 400, 200, or 100 mg/kg/day, respectively). Treatment was administered for 15 consecutive days in the plateau environment. The 10 rats in the normal control group received normal saline (1 mL/100 g/day) for 15 consecutive days in a plain environment.

All animal handling procedures used in this study were approved by the Institutional Animal Care and Use Committee of Xinjiang Medical University (approval number: IACUC-20210303-49).

### Cell culture, modeling, and grouping

PAECs were cultured in a system containing 10% FBS, 1% penicillin‒streptomycin, and 1% cell growth factor. The cells were subcultured when they reached 70–80% confluence. The cells were divided into five groups: a normal control group, a model group that was incubated for 1 h with 800 µM H_2_O_2_, and three other groups that were incubated with TTR (20, 40, or 80 µg/mL, respectively) followed by incubation for 1 h with 800 µM H_2_O_2_ 24 h after the intervention.

### Determination of PAP

Twenty-four hours after drug administration, the rats in each group were anesthetized with 3% pentobarbital sodium by intraperitoneal injection and fixed on an operating table in a supine state. Pulmonary artery catheterization was used to measure the PAP of the animals. The subcutaneous tissues were bluntly separated from the muscles by cutting the skin on the right side of the neck, and the carotid artery was dissociated and clearly exposed. A small slit was cut in the artery, and a polyethylene catheter containing a pressure sensor was slowly pushed into the carotid artery on the right side of the neck and then into the pulmonary artery via the ventriculus dexter. Changes in the PAP waveform (mPAP) were recorded.

### Histopathology of pulmonary vessel structure and morphology

Lung tissues were collected from each group of rats and fixed in 10% formaldehyde solution. The specimens were placed in 80% ethanol and then in 95% ethanol, dehydrated in absolute ethyl alcohol, and placed in xylene until they became transparent. The specimens were embedded in paraffin after becoming transparent. The tissues were cut into 5-µm slices, dewaxed with xylene, and dehydrated in an ethanol gradient series. The slices were stained with hematoxylin, differentiated with 1% HCl-ethanol, stained with 1% weak aqua-ammonia and eosin, dehydrated in a graded ethanol series, and sealed with neutral balsam mounting medium. Each stained slice was observed under a light microscope, and 25–30 pulmonary arterioles (diameter: 300–500 μm) were randomly selected for evaluation. Remodeling and proliferation of the pulmonary arterioles in the lungs were determined based on vessel wall thickness (WT%), which was calculated using the following formula:

WT% = (outer diameter of pulmonary arteriole - inner diameter of pulmonary arteriole)/(outer diameter of pulmonary arteriole) × 100%.

### Determination of SOD, GSH-Px, and MDA levels in each group of rats with HAPH

Blood samples were collected from the pulmonary artery of each animal and centrifuged for 20 min at 4 °C and 3000 r/min. The precipitate was removed, the serum was isolated, and the activities of SOD and GSH-Px and the concentration of MDA were determined by ELISA. The assays were performed in strict compliance with the instructions provided in the kits.

### Determination of cell viability using CCK-8 assays

Cells in logarithmic growth were inoculated into 96-well culture plates and allowed to adhere by incubating them overnight in culture medium. The cells were subjected to drug treatment for 24 h followed by treatment with H_2_O_2_ for 1 h, and the primary culture medium was then discarded. Approximately 100 µL of culture medium containing CCK-8 was then added to each well, and the plates were incubated for 2 h. The optical density (OD) of each well at a wavelength of 450 nm was determined by ELISA, and the cell viability was calculated (cell viability = [(OD_experimental well_-OD_blank well_)/(OD_control well_-OD_blank well_)] × 100%).

### Determination of apoptosis by flow cytometry

Cells in logarithmic growth were inoculated into six-well culture plates and cultured overnight. After treatment of the cells with drugs for 24 h followed by treatment with H_2_O_2_, for 2 h, the primary culture medium was discarded. An annexin V-FITC/PI apoptosis assay kit was used for staining, and the rate of apoptosis of the cells in each group was determined by flow cytometry (FCM).

### Determination of ROS levels in cells by FCM

Cells in logarithmic growth were inoculated into six-well culture plates and cultured overnight. After treatment of the cells with drugs for 24 h and with H_2_O_2_ for 2 h, the primary culture medium was discarded. A DCFH-DA fluorescent probe was then used to stain the cells, and ROS levels in the cells were measured using FCM.

### Western blotting

Tissues and cells were ground or lysed, respectively, with RIPA lysis buffer and centrifuged, and the supernatants were collected. The proteins were quantified by the BCA method, mixed with loading buffer, denatured for 10 min in a water bath at 100 °C and stored at − 20 °C. The denatured proteins were separated using sodium dodecyl sulfate‒polyacrylamide gel electrophoresis and transferred to polyvinylidene fluoride membranes, which were then incubated in 5% skim milk and sealed for two hours at room temperature. After sealing, the membranes were incubated at 4 °C overnight with antibodies against Nrf2 (1:500), HO-1 (1:1000), Bax (1:2000), Bcl-2 (1:2000), or *β*-actin (1:20,000); the corresponding secondary antibodies were then added, and the membranes were incubated for an additional hour at room temperature. After enhanced chemiluminescence development, the levels of expression of individual proteins were analyzed using ImageJ software and expressed in relative terms.

### Statistical analysis

SPSS22.0 was used for data analysis, and GraphPad Prism 8.2 was used to prepare graphs. All experimental data are expressed as mean and standard deviation. One-way ANOVA was employed for between-group comparisons, and an α of 0.05 indicated a significant difference.

## Results

### Effects of TTR on mPAP in rats

**Fig. 1:**
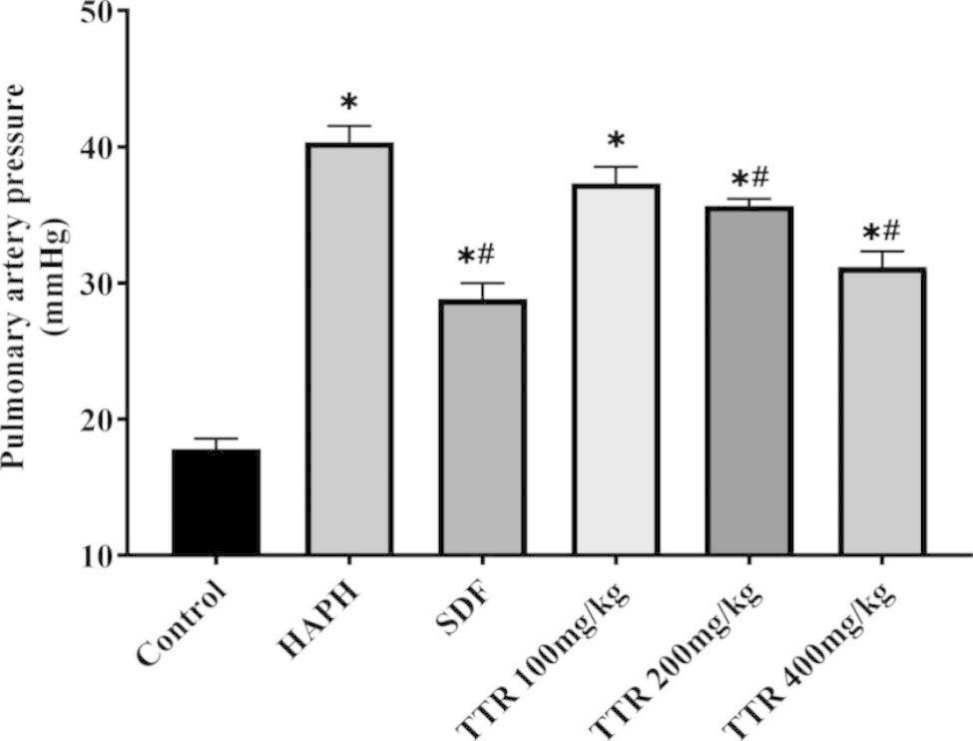
Effects of TTR on mPAP in rats

The mPAP of the HAPH model group increased markedly compared with that of the plain model group. The mPAP of HAPH rats to which sildenafil was administered clearly decreased, suggesting that the method adopted in the present study can be used to establish a stable rat model of HAPH. In the groups that received TTR, mPAP was reduced to varying degrees; the reduction was the greatest in the TTR-H group, indicating that administration of TTR can alleviate HAPH (Fig. 1).

### Pathological changes in the lung tissues of rats in each group

**Fig. 2:**
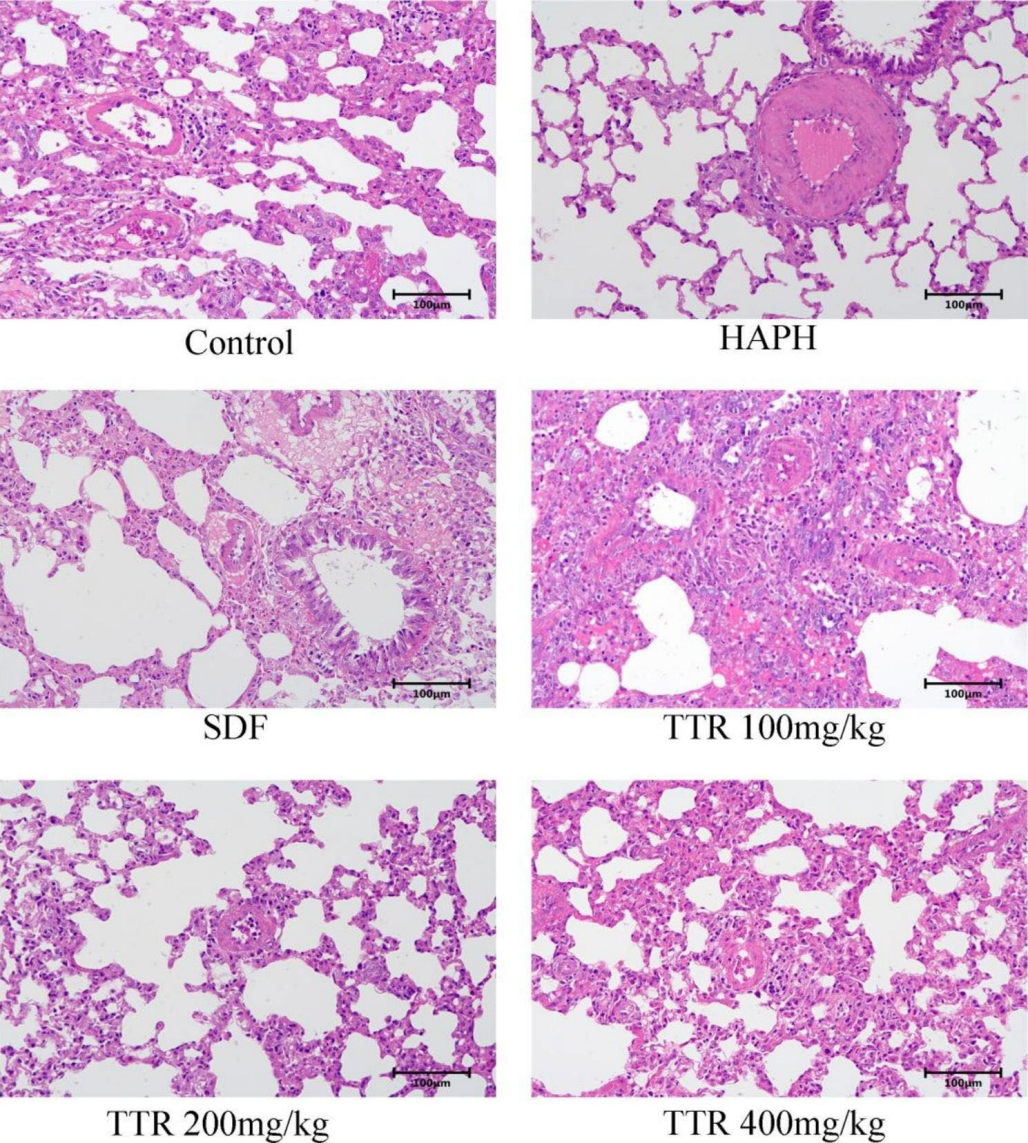
Hematoxylin and eosin staining of small pulmonary arteries in each group of rats

**Fig. 3:**
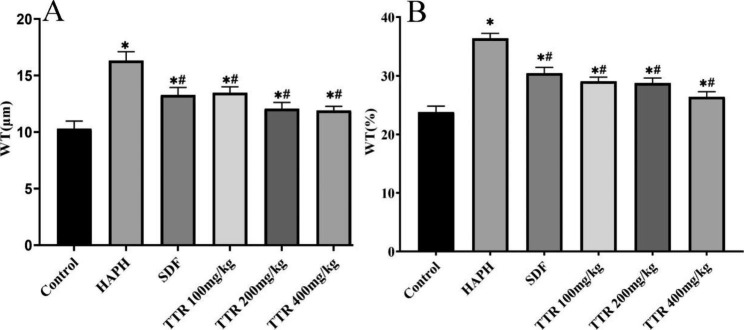
TTR effectively alleviates pulmonary vascular remodeling in HAPH rats. (**A**) graph showing pulmonary vascular wall thickness; (**B**) graph showing pulmonary vascular wall thickness percentage;

Compared with the plain model group, the WT% of the lung tissues of rats in the HAPH model group increased markedly, indicating that high-altitude hypoxia caused pulmonary vascular remodeling in animals with HAPH. After administration of TTR at any of the doses tested, the WT% of the pulmonary vessels of the rats decreased, indicating that TTR can alleviate or slow pulmonary vascular remodeling induced by high-altitude hypoxia (Figs. 2 and 3).

### Changes in the concentrations of MDA, SOD, and GSH-Px in the sera of animals

Compared with the plain model group, SOD and GSH-Px activity in the sera of the rats in the HAPH model group decreased to varying degrees, and MDA levels increased to varying degrees (*P* < 0.05), indicating that high-altitude hypoxia subjected HAPH rats to oxidative stress. SOD and GSH-Px activity in the sera of each group that received TTR increased to varying degrees, and MDA levels decreased to varying degrees (*P* < 0.05), indicating that TTR can increase the oxidative stress level in the rats (Table 1).

**Table 1:** Changes in the concentrations of MDA, SOD, and GSH-Px in the sera of animals

Group	GSH-Px (U)	SOD(U/mL)	MDA(µmol/L)
Control	7415.38 ± 132.97	290.23 ± 26.75	7.51 ± 1.43
HAPH	4411.72 ± 138.29*	336.42 ± 23.87*	11.32 ± 2.05*
TTR 100 mg/kg	6741.39 ± 184.58*#	311.85 ± 24.69*	10.80 ± 1.74*#
TTR 200 mg/kg	7124.91 ± 198.30*#	302.87 ± 19.95*#	9.30 ± 2.08*#
TTR 400 mg/kg	7205.09 ± 180.15*#	299.09 ± 27.85*#	8.87 ± 1.88#

Note: Compared with Control, *P<0.05; compared with HAPH, #*P*<0.05

### Changes in the expression of Nrf2, HO-1, bax, and Bcl-2 in rat lung tissues

**Fig. 4:**
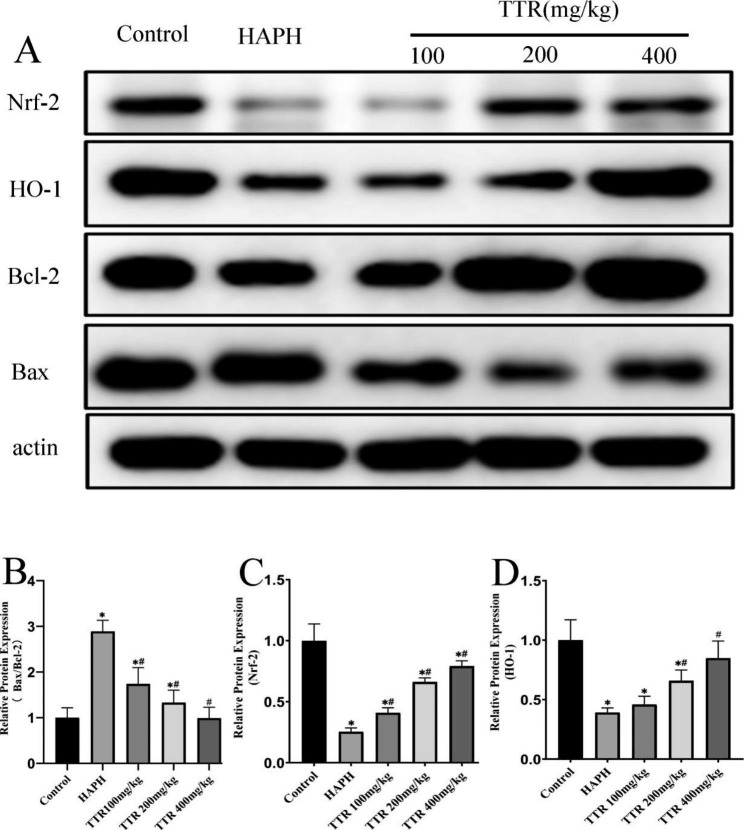
Effect of TTR on the protein expression of Nrf-2, HO-1, Bax, and Bcl-2 in the lung tissue of HAPH rats

The expression of Bax in the lung tissues of the HAPH model group was unchanged compared with that of the plain model group, whereas the expression of Bcl-2, Nrf2, and HO-1 was downregulated in the model group (*P* < 0.05). The expression of Bax in the groups treated with TTR at low, medium, and high doses was downregulated compared with that of the model group, whereas the expression of Bcl-2, Nrf2, and HO-1 was upregulated in the TTR-treated groups (*P* < 0.05). This result suggested that TTR has strong antagonistic actions against apoptosis-induced thoracic injury and that it exerts a regulatory effect on oxidative stress (Fig. 4).

### Changes in the viability of PAECs in rats

**Fig. 5:**
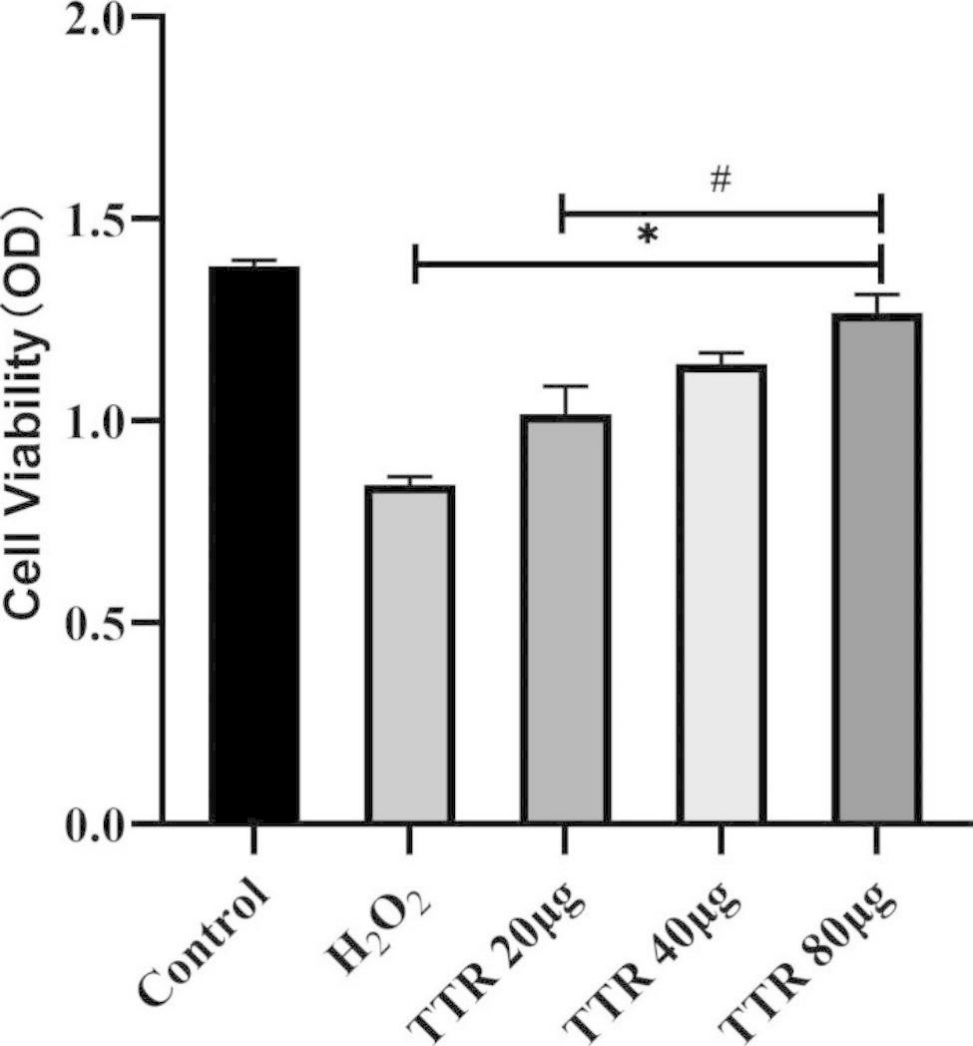
Results of the CCK-8 cell viability assay

Compared with the normal control group, PAEC viability after H_2_O_2_ treatment clearly decreased (*P* < 0.05). Cell viability after drug intervention increased in a dose-dependent manner compared with the viability of the model group (*P* < 0.05). This result suggests that TTR exerts protective effects against H_2_O_2_-induced damage in PAECs (Fig. 5).

### Effects on apoptosis

**Fig. 6:**
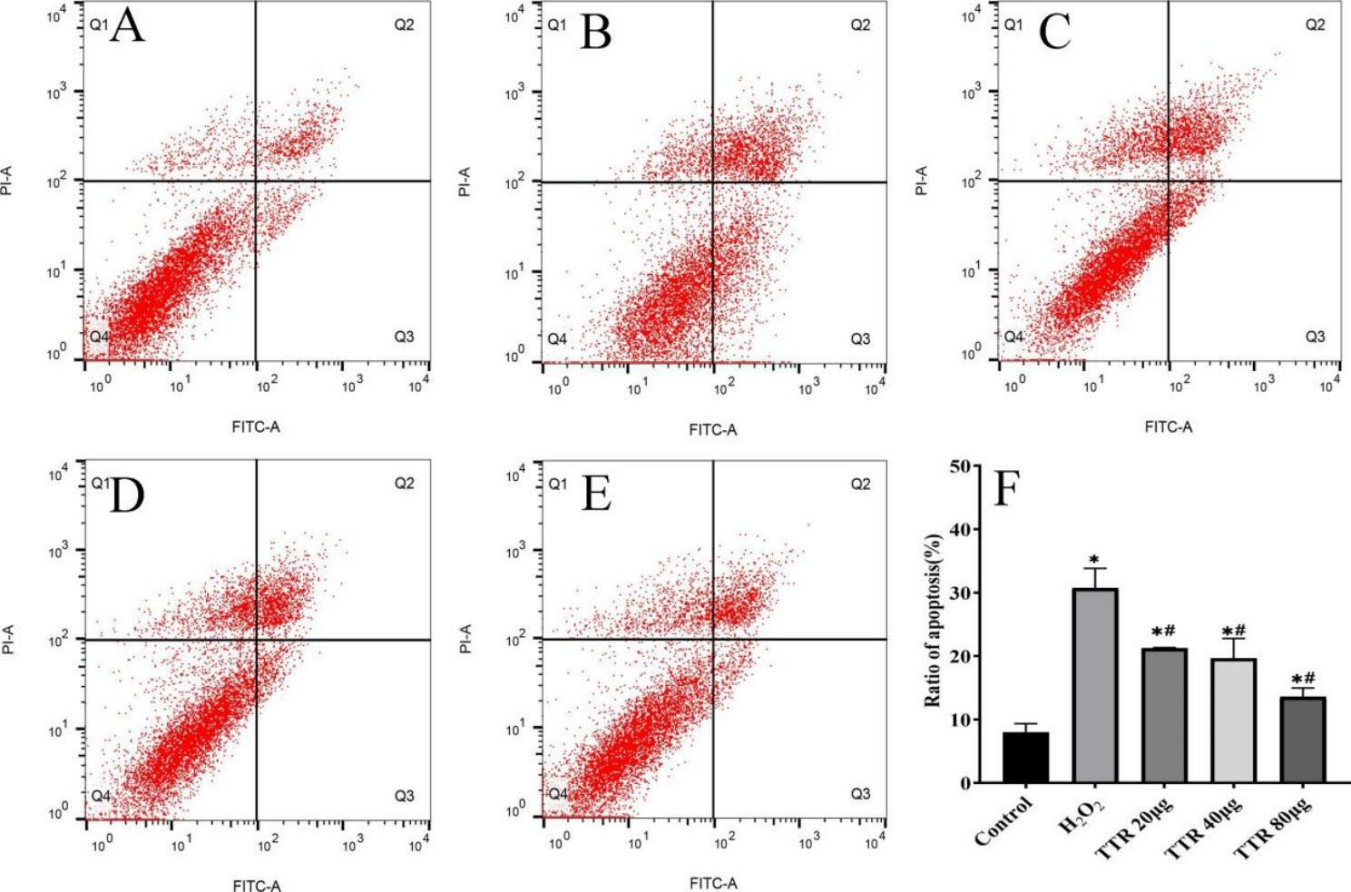
TTR inhibits H_2_O_2_-induced endothelial cell injury in rat pulmonary arteries. (**A**) control group; (**B**) H_2_O_2_ group; (**C**) TTR 20 µg; (**D**) TTR 40 µg; (**E**) TTR 80 µg; (**F**) results of the apoptosis analysis of PAECs

The apoptosis rate of the model group was markedly increased compared with that of the normal control group. The apoptosis rate of the TTR-treated group clearly decreased compared with that of the model group, and the difference was statistically significant (*P* < 0.05). This result suggests that TTR has strong antagonistic action against H_2_O_2_ damage to PAECs (Fig. 6 ).

### Effects on ROS levels in cells

**Fig. 7:**
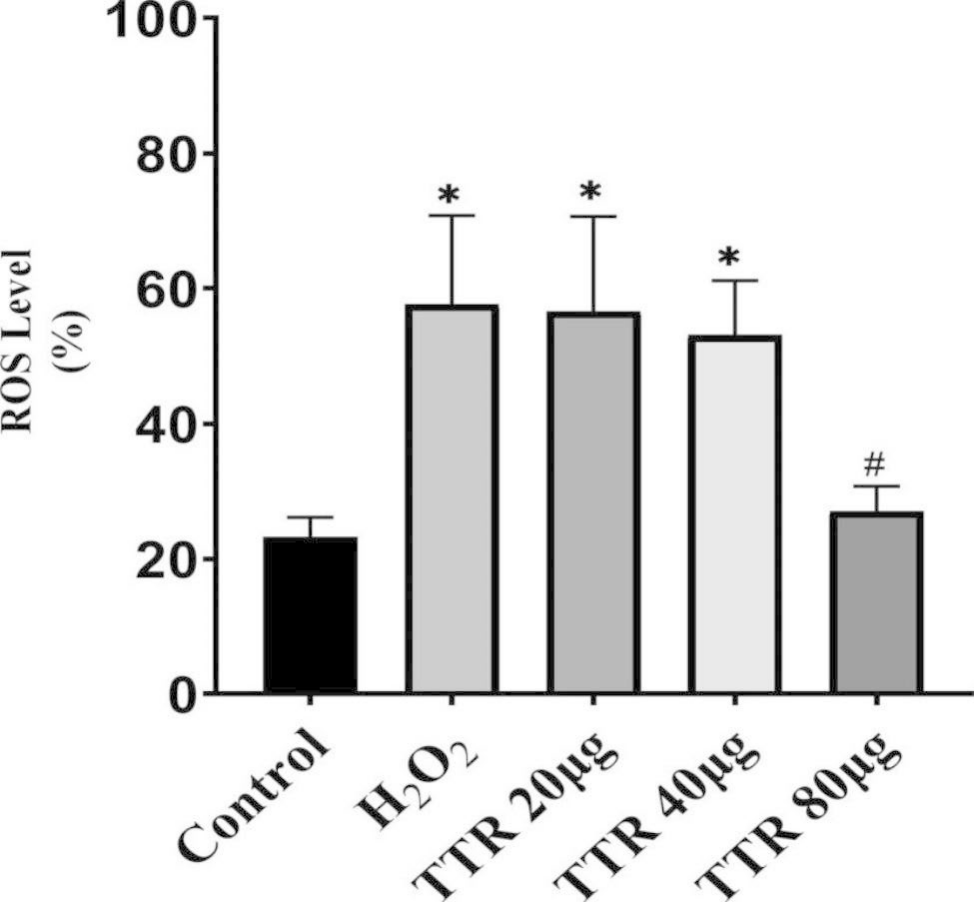
Results of the ROS assay

The level of ROS in the H_2_O_2_ model group was elevated compared with that in the normal control group. After drug intervention, the ROS levels in the cells gradually declined with increasing drug dose and concentration. This result suggests that TTR decreases the level of ROS in PAECs that have been exposed to H_2_O_2_ (Fig. 7).

### Effects on the expression of Nrf2, HO-1, bax, and Bcl-2 in PAECs

**Fig. 8:**
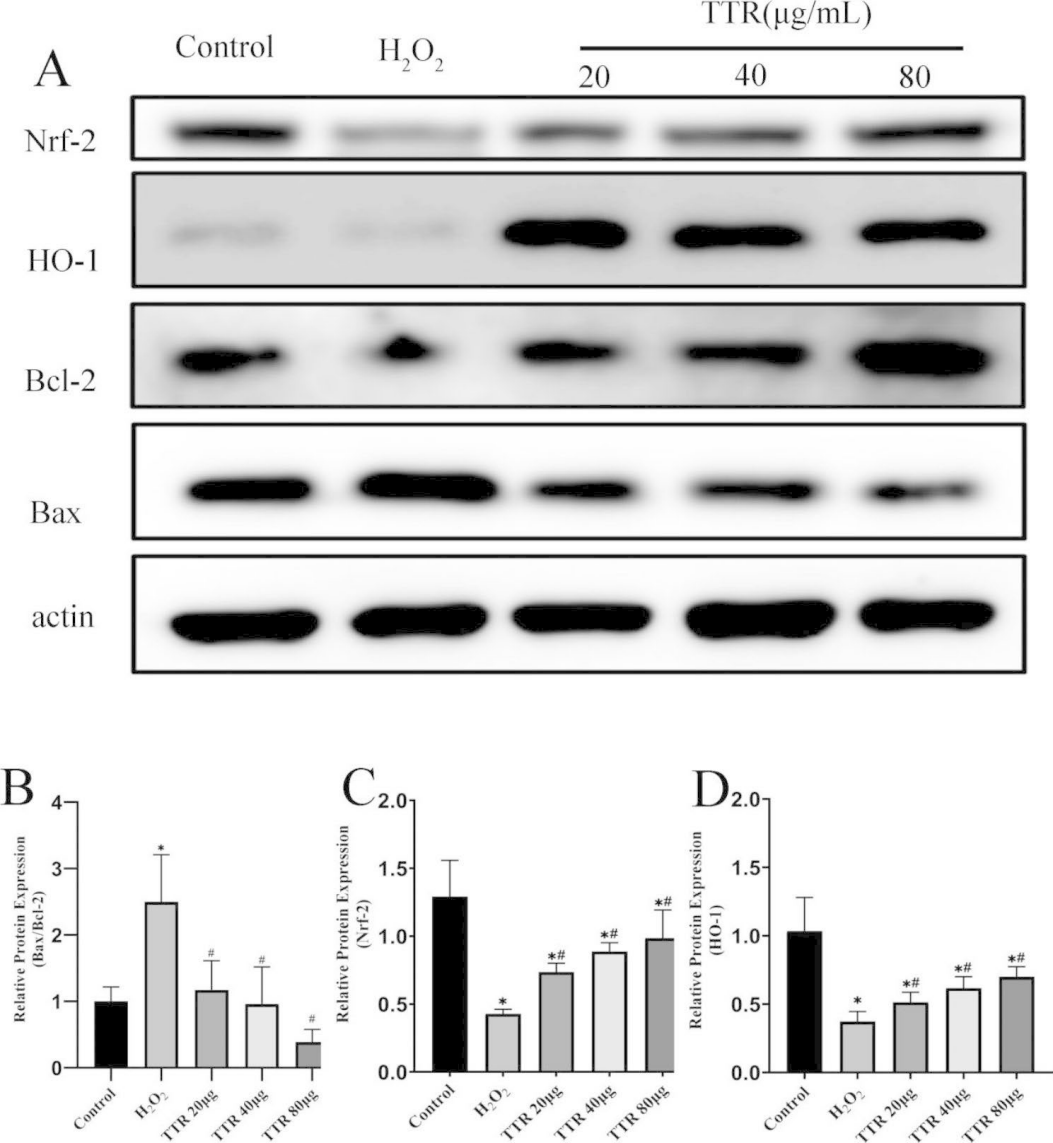
Effect of TTR on the levels of Nrf-2, HO-1, Bax, and Bcl-2 proteins in rat PAECs.

The expression of Bax in the model group was upregulated compared with that in the normal control group, whereas the expression of Bcl-2, Nrf2, and HO-1 was downregulated in the model group. The expression of Bax in the treated group was downregulated compared with that in the model group, and the expression of Bcl-2, Nrf2, and HO-1 was upregulated in the treated group. This result suggests that TTR has strong antagonistic actions against H_2_O_2_-induced apoptosis in PAECs and that it regulates oxidative stress via the Nrf2/HO-1 pathway (Fig. 8).

## Discussion

HAPH is chronic and persistent and has a slow onset. Its early clinical symptoms are atypical and nonspecific. Missed and delayed diagnoses often occur due to a lack of clinical diagnostic awareness. Morbidity and mortality due to the condition increase with altitude. HAPH seriously affects the quality of life of patients and threatens their lives [2]. The drugs that have been clinically approved for the treatment of pulmonary hypertension mainly target three key pathways: the endothelin pathway, the prostacyclin pathway, and the nitric oxide/soluble guanylate cyclase pathway [27]. Administration of these drugs can reduce the tension within the pulmonary artery, thereby improving symptoms to some extent, but they cannot prevent or reverse the progression of the disease [28]. Through the establishment and use of rat models of HAPH and H_2_O_2_-induced PAEC damage, the present study found that TTR exerts a protective effect in HAPH rats by regulating the Nrf2/HO-1 signaling pathway. The results of our experiments showed that the PAP of the animals in the plateau model group was elevated, whereas the PAP of the animals in the plateau model group to which sildenafil was administered decreased markedly, indicating that a rat model of HAPH had been successfully generated. After administration of various doses of TTR, the mean PAP of the animals in the model group decreased markedly, and the WT% and WA% declined dramatically, indicating that TTR lowered PAP in rats. The in vitro experimental results showed that the activities of PAECs were clearly inhibited after H_2_O_2_ treatment. The PAECs that were treated with TTR showed marked alleviation of H_2_O_2_-induced oxidative damage, indicating that TTR has certain protective effects on H_2_O_2_-induced damage to PAECs.

Oxidative stress is a key factor in the occurrence and development of pulmonary hypertension [29, 30]. Oxidative stress can occur due to an increase in the production of ROS or to exhaustion of the antioxidant system [31–33]; either of these conditions can lead to an imbalance between antioxidants and pro-oxidants. When exposed to a high-altitude hypoxic environment, the body develops an oxidative stress reaction that induces ROS overload [34–36]. ROS attack polyunsaturated fatty acids in biofilms and trigger a lipid peroxidation chain reaction, resulting in biofilm dysfunction and damage to enzymes that are located within membranes [37]. High levels of ROS can also lead to mitochondrial damage, DNA modification, increased production of cytokines, and even cell death [38–40]. ROS can also induce redox signaling pathways that promote pulmonary vascular smooth muscle proliferation and lead to vascular remodeling [41–43]. Blocked synthesis and decreased activity of NO derived from endothelial nitric oxide synthase (eNOS) are important factors in the occurrence of vascular endothelial function damage [44]. Oxidative stress can inhibit the activity of eNOS, reduce the level of NO in blood vessels, and affect pulmonary vascular endothelial function. GSH-Px and SOD are the key enzymes that scavenge ROS in vivo. If the activity of ROS scavengers decreases, the number of oxygen free radicals will increase excessively, resulting in damage to the lipids within cell membranes and catalysis of arachidonic acid to MDA, an indicator of lipid peroxidation [38, 45]. The in vivo and in vitro experimental results obtained in this study showed that the MDA level in the model group was significantly increased and that the activities of SOD and GSH-Px were significantly decreased. The TTR-treated groups had MDA levels that were decreased to varying degrees and SOD and GSH-Px activities that were increased to varying degrees, indicating that TTR treatment alleviated the oxidative stress injury that otherwise occurs in HAPH.

Apoptosis is also known as programmed cell death [46]. Studies have confirmed that HAPH is related to an imbalance between apoptosis and cell proliferation and that apoptosis of pulmonary vessel endothelial cells plays a crucial role in the onset of HAPH. Apoptosis of endothelial cells may cause endothelial dysfunction, endocrine dysfunction, and an imbalance between vasorelaxation and vasoconstriction factors, thereby elevating pulmonary arterial systolic pressure [47]. Moreover, apoptosis may impair endothelial barrier function, cause morphological and structural changes in blood vessels, dilate intercellular spaces, and facilitate interaction between growth factors and PASMCs via the intercellular space, thereby promoting the proliferation and migration of PASMCs and increasing intima media thickness and pulmonary vascular remodeling [9]. The *BCL2* and *BAX* genes, which belong to the Bcl-2 family, are the most important known regulatory genes in apoptosis. Bax promotes apoptosis, whereas Bcl-2 inhibits it. An imbalance between the two may induce apoptosis [46]. The in vitro and in vivo experimental results obtained in the present study showed that Bax expression was elevated in the model group, whereas Bcl-2 expression decreased; as a result, the Bax/Bcl-2 ratio increased. In the treated group, in contrast, Bax expression was downregulated and Bcl-2 expression was upregulated, indicating that TTR had inhibitory effects on apoptosis in PAECs.

To ensure oxygen saturation of the blood at the high elevation of the plateau, the amount of hemoglobin in the blood increases [48]. Hemoglobin is a scavenger of NO, and it can reduce the bioavailability of NO [49] and react with hyperoxide and hydrogen peroxide to produce ROS-damaged cells. Endothelial and smooth muscle cells are vulnerable to hyperoxide-induced damage; cells that are damaged in this way exhibit cellular dysregulation that induces vasoconstriction and elevates blood pressure through smooth muscle contractions. Heme oxygenase (HO) is a rate-limiting enzyme in heme metabolism, and HO-1, also known as inducible HO, can be induced as a protective mechanism when animals are exposed to oxidative stress, heme, endotoxins, cytokines, or other exogenous factors. Studies have confirmed that HO-1 protects cells from oxidative stress and that it acts as a catalyst in the oxidation of heme to biliverdin, carbon monoxide, and ferrum (Fe^2+^); in this way, it alleviates the cytotoxicity of heme, decomposes its product biliverdin, and exerts a strong antioxidant function [50]. The findings of the present study showed that TTR treatment reduced ROS levels in PAECs that had been damaged by H_2_O_2_. The western blotting results obtained in the in vitro and in vivo experiments showed that the expression of Nrf2 and HO-1 was downregulated in the model group but that it was upregulated after treatment with TTR. This result suggests that TTR can play a role in antioxidative stress by regulating the expression of Nrf2 and HO-1.

Our study explores the effects and molecular mechanisms through which TTR alleviates pulmonary hypertension from both the in vivo and the in vitro perspectives. However, due to various factors, our research also has certain limitations. We did not employ Nrf2/HO-1 pathway-related agonists, inhibitors, siRNA interference, or certain other methods in our study.

## Conclusions

TTR reduced mPAP in rats with HAPH, alleviated or slowed pulmonary vascular remodeling, inhibited ROS production, and alleviated oxidative stress. It produces these effects through regulation of the Nrf2/HO-1 signaling pathway. TTR shows great potential for use in HAPH treatment.

## Electronic supplementary material

Below is the link to the electronic supplementary material.

Additional file 1

## Data Availability

The datasets used and/or analyzed during the current study are available from the corresponding author on reasonable request.

## Abbreviations

BAX: BCL-2-associated X protein
BCL2: B-cell lymphoma-2
GSH-Px: Glutathione peroxidase
HAPH: High-altitude pulmonary hypertension
HO-1: Oxygenase1
MDA: Malondialdehyde
mPAP: Mean pulmonary arterial pressure
Nrf2: Nuclear factor E2-related factor 2
PAECs: Pulmonary artery endothelial cells
ROS: Reactive oxygen species
SOD: Superoxide dismutase
TTR: Tannin of *Terminalia bellirica* (Gaertn.) Roxb.
