# Supplementary material for: Protective effects of the Terminalia bellirica tannin-induced Nrf2/HO-1 signaling pathway in rats with high-altitude pulmonary hypertension
Source: BMC Complement Med Ther. 2023 May 6;23:150. doi: 10.1186/s12906-023-03981-2 (PMC10163731; doi:10.1186/s12906-023-03981-2)
Supplement: Supplementary file 1 — Additional file 1 [file 12906_2023_3981_MOESM1_ESM.doc]

**Supplementary Information**

**Supplementary Figure 2. Original western blotting for Nrf-2, HO-1, Bax, and Bcl-2 proteins in the lung tissue of HAPH rats.**

| ***β*-actin** | **Bax** |
| --- | --- |
| 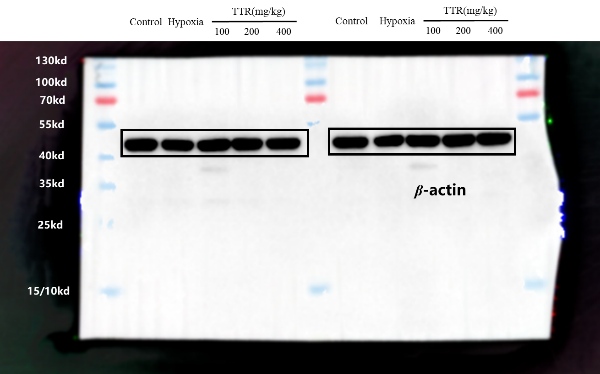 | 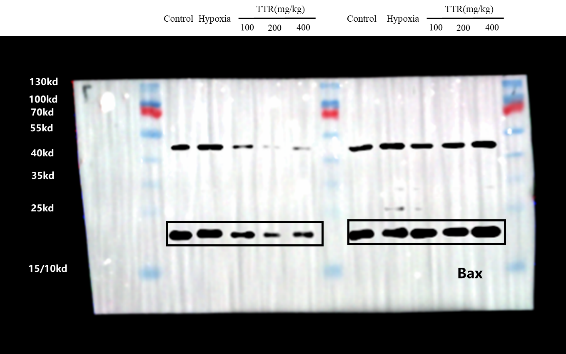 |
| **Bcl-2** | **HO-1** |
| 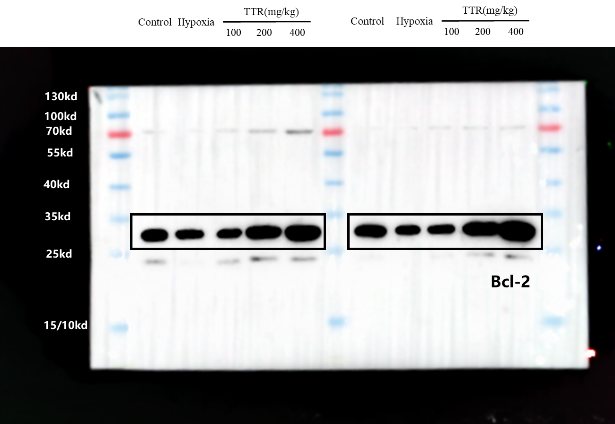 | 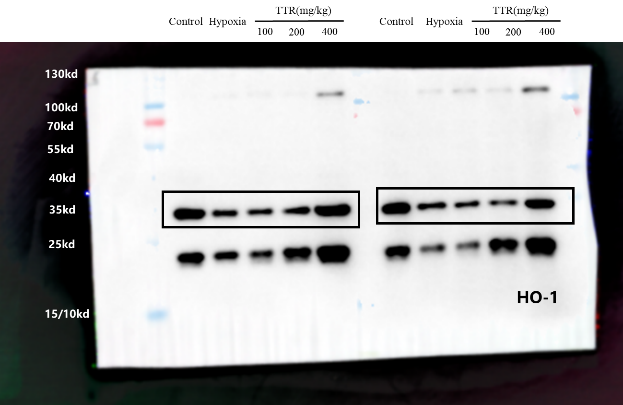 |
| **Nrf-2** |  |
| 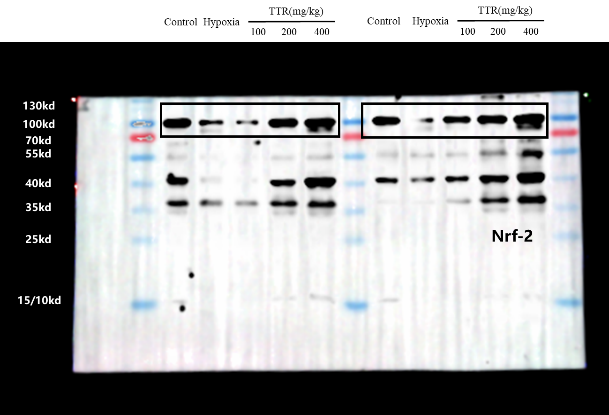 |  |

**Supplementary Figure 4. Original western blotting for protein from Nrf-2, HO-1, Bax, and Bcl-2 proteins in rat PAEC cells**

| ***β*-actin** | **Bax** |
| --- | --- |
| 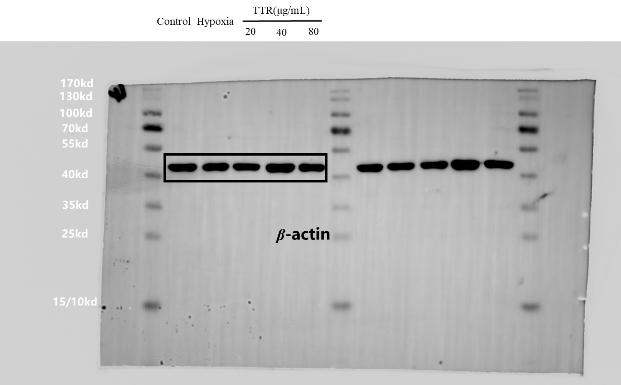 | 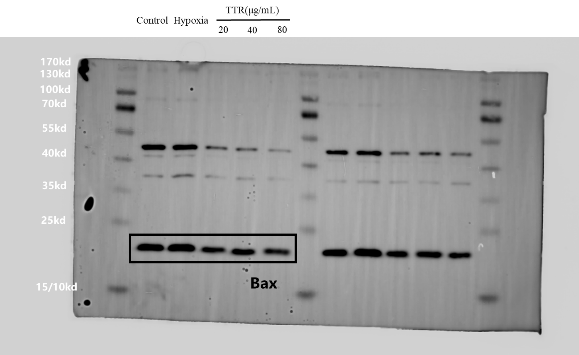 |
| **Bcl-2** | **HO-1** |
| 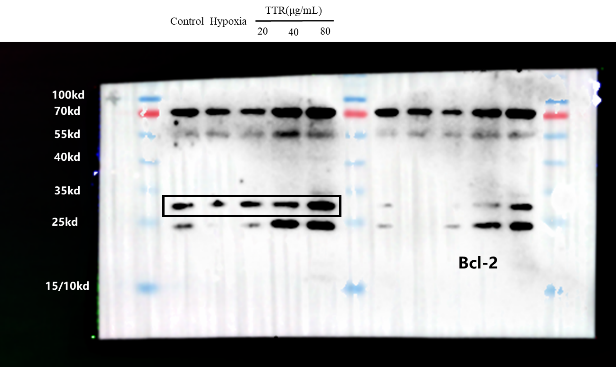 | 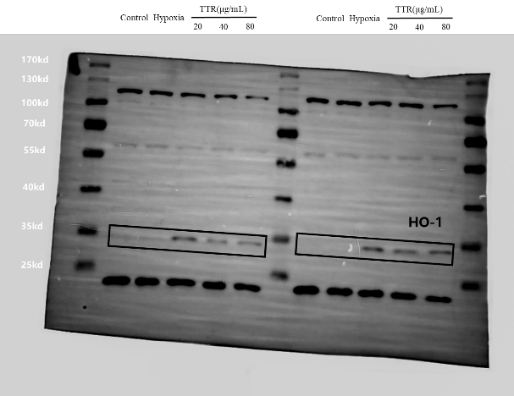 |
| **Nrf-2** |  |
| 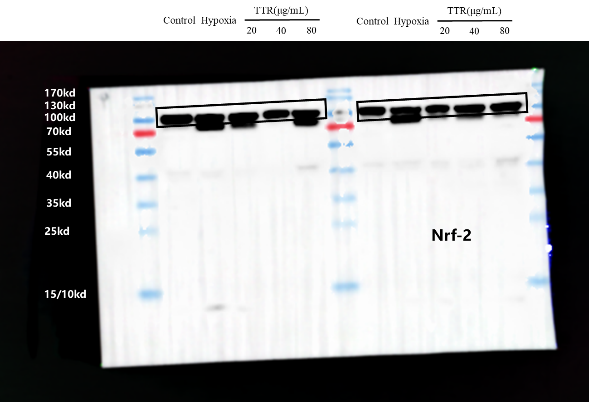 |  |
